# Performance Validation of COVID-19 Self-Conduct Buccal and Nasal Swabs RTK-Antigen Diagnostic Kit

**DOI:** 10.3390/diagnostics11122245

**Published:** 2021-11-30

**Authors:** Mohammad Nur Amin Kalil, Wardah Yusof, Naveed Ahmed, Mohd Hashairi Fauzi, Mimi Azliha Abu Bakar, Afifah Sjamun Sjahid, Rosline Hassan, Chan Yean Yean

**Affiliations:** 1 Department of Emergency Medicine, Hospital Universiti Sains Malaysia, Universiti Sains Malaysia, Kubang Kerian 16150, Kelantan, Malaysia; aminkalil28@student.usm.my (M.N.A.K.); hashairi@usm.my (M.H.F.); azliha79@usm.my (M.A.A.B.); afifahkk@usm.my (A.S.S.); 2 Department of Medical Microbiology and Parasitology, School of Medical Sciences, Universiti Sains Malaysia, Kubang Kerian 16150, Kelantan, Malaysia; wardahyusof@usm.my (W.Y.); naveed.malik@student.usm.my (N.A.); 3 Department of Hematology, School of Medical Sciences, Universiti Sains Malaysia, Kubang Kerian 16150, Kelantan, Malaysia; roslin@usm.my

**Keywords:** performance validation, SARS-CoV-2, rapid self-conduct RTK-antigen test

## Abstract

The antigen rapid diagnostic test (Ag-RDT) is an immunodiagnostic test that detects the presence of viral proteins (antigens) expressed by the COVID-19 virus in a sample from a patient’s respiratory tract. This study focused on evaluating the performance of self-conduct buccal and nasal swabs RTK-antigen test compared to nasopharyngeal swab RTK-based COVID-19 diagnostic assays, Panbio™ COVID-19 Ag Rapid Test Device (Nasopharyngeal) (Abbott Rapid Diagnostics Jena GmbH, Jena, Germany) used in hospitals for first-line screening. The sensitivity and specificity of the paired RTK-Ag test in detecting the an-tigen were calculated at 96.4% and 100%, respectively. Fisher exact tests showed the association between nasopharyngeal swabs RTK-Ag assay and buccal-nasal swabs RTK-Ag from Prodetect^TM^ is significant (*p*-values < 0.001). The result showed that a self-conducted buccal and nasal RTK-antigen rapid test by the patients is comparable to the results obtained from a rapid test device conducted by trained medical personnel using a nasopharyngeal swab.

## 1. Introduction

COVID-19 is an infectious disease caused by the SARS-CoV-2 virus. The clinical spectrum of the disease is heterogeneous, ranging from asymptomatic to severe respiratory disease and death commonly manifested with fever, cough, loss of sense of smell and shortness of breath [1]. Diagnoses are made from nasopharyngeal swab specimens and the virus is identified using quantitative reverse transcription-PCR (RT-qPCR) assays performed according to World Health Organization (WHO) guidelines to detect SARS-CoV-2. However, detecting antibodies for previous exposure cases through rapid test kit (RTK)-antibody rapid lateral flow assays is uncertain for seroprevalence studies [2,3].

Analysis of RT-qPCR utilizing nasopharyngeal swabs, throat swabs, or saliva is the gold standard for COVID-19 diagnosis [4,5]. There are also developed RT-qPCR kits that do not need viral RNA extraction and high-throughput RT-qPCR equipment [3]. Although such tests are commonly used in public health laboratories and larger, well-equipped hospitals, they are not accessible in small clinics. Consequently, human samples are transferred to facilities with RT-qPCR capacity, delaying test results for suspected COVID-19 patients [1]. The expensive and time-consuming RT-qPCR also necessitates specialized equipment and well-trained laboratory staff [6].

In response to the continuously increasing SARS-CoV-2 infections and shortage of molecular testing capacity, biotechnology companies and diagnostic test manufacturers have been developing and selling different types of rapid diagnostic tests (RDTs) for use outside of laboratory settings as a home-based kit [7,8]. The RDTs can be based on protein (antigen) to detect active SARS-CoV-2 infections from respiratory samples [9] or to detect human antibodies in our immune system generated in response to SARS-CoV-2 infection [10].

The RDTs are user-friendly, cost-effective, and safe point-of-care testing (POCT); however, real-time performance and validation of these assays is still a topic of concern [11,12]. The nasopharyngeal swab technique is invasive and may result in bleeding, with a higher risk of SARS-CoV-2 transmission to healthcare personnel [13]. On the other hand, saliva, buccal, and nasal swab collection are noninvasive and may be done safely outside hospitals. In addition, compared to nasopharyngeal swabs, self-collection of these types of samples may decrease the risk of SARS-CoV-2 transmission to healthcare personnel [14,15].

Ag-RDTs for COVID-19 was authorized for clinical usage in Malaysia and other countries, and their sensitivity was compared to that of various types of RT-qPCR [6,16]. Although these Ag-RDTs may help identify COVID-19 patients in a short time, their sensitivity is critical in deciding how the community should utilize them.

The current study helps understand the performance evaluation of self-conduct buccal and nasal swabs RTK-antigen test to generate knowledge for current and long-term control and management outbreak in Malaysia. This research study focused on evaluating the performance of self-conduct buccal and nasal swabs RTK-antigen test compared to nasopharyngeal swab RTK-based COVID-19 diagnostic assays routinely used in hospitals for first-line screening.

## 2. Materials and Methods

### 2.1. Study Design and Settings

This evaluation study was conducted in the Emergency Department (ED) of Hospital Universiti Sains Malaysia (HUSM), Kelantan, Malaysia, from 13 August to 20 September 2021. This study was conducted under the ethical approval of the Human Research Ethics Committee of Universiti Sains Malaysia (USM) (JEPeM) (Ethical Approval No: USM/JEPeM/COVID19-44, approved on 19 July 2020).

### 2.2. Patients’ Recruitment

A total of 120 patients presenting with signs and symptoms of COVID-19 were recruited to evaluate the specificity, sensitivity and accuracy of COVID-19′s antigen self-kit using a buccal–nasal swab, Prodetect^TM^ (Mediven Sdn Bhd, Penang, Malaysia). Before recruiting the patients, informed consent was obtained, and patients were tutored on the self-testing protocol. Inclusion criteria: (1) No age and gender limit on patients’ recruitment; (2) Only patients diagnosed with COVID-19 infection using nasopharyngeal swab antigen detection kit were recruited for this study.

### 2.3. Clinical Sample Collection

Each patient was asked to conduct the Prodetect^TM^ using concurrent two types of samples (buccal and nasal swabs) by themselves. In order to validate the accuracy of the test protocol, the testing protocol was observed by trained medical personnel. The sample collection and disposal of the kit are in accordance with the standard operating procedures (SOPs) for COVID-19 as recommended by World Health Organization (WHO). The patients’ COVID-19 diagnosis was counter-checked from Malaysia’s Public Health Laboratory Information System (SIMKA) database (https://simka.moh.gov.my/, accessed on 1 October 2021).

### 2.4. Buccal–Nasal Prodetect^TM^ RTK-Ag Self-Test

#### 2.4.1. Prodetect^TM^ Buccal–Nasal Test Principle

Prodetect^TM^ COVID-19 Antigen rapid self-test is a qualitative membrane-based immunoassay to detect SARS-CoV-2 nucleocapsid protein antigens in the human buccal and nasal specimen. During the test, the specimen will react with SARS-CoV-2 nucleocapsid protein antibody-coated particles in the line region of the test device. The mixture migrates upward on the membrane chromatographically by capillary action and reacts with SARS-CoV-2 nucleocapsid protein antibody in the test line region. If the specimen contains SARS-CoV-2 antigens, a colored line appears in the test line region. If the specimen does not contain SARS-CoV-2 antigen, a colored line will not appear in the test line region, indicating a negative result. A colored line at the Control region serves as a procedure control, indicating that the test has been performed correctly.

#### 2.4.2. Self-Test Specimen Collection Procedure

For buccal swabs, before collecting saliva, the participant was asked to not place anything into his/her mouth, including food, drink, or tobacco products, for at least 10 min before sample collection. A sterile swab was used to rub up and down the oral cavity at least five times on both cheeks before the swab was placed and mixed carefully with the buffer in the buffer tube.

For collecting the nasal swab specimen, the tip of a new sterile swab was inserted into the left and right nostril until a slight resistance (about 2 cm of the nose) was felt and gently rotated 5–10 times against the nasal wall. The swab was removed from the nasal cavity and mixed with the buffer to make an extraction sample. After the sample collection, both of the swabs were discarded according to the standard waste disposal guidelines.

#### 2.4.3. Antigen Detection Using Rapid Self-Test Lateral Flow Chromatographic Immunoassay

**Figure 1:**
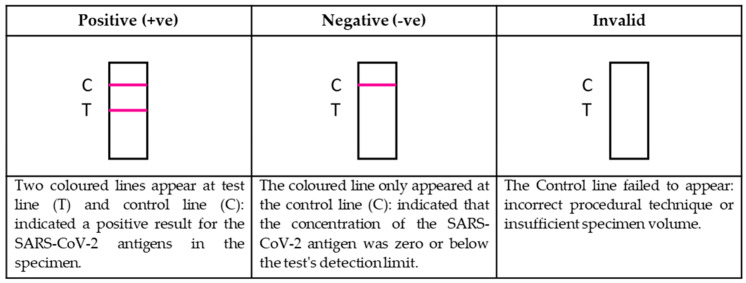
Interpretation and Reporting of Results.

Immediately after specimens collection, the test was performed by putting two drops of the extraction sample on the test device and incubated for 15 min at room temperature. The results appeared as colored lines, interpreted and reported based on a lateral flow mechanism, as shown in Figure 1.

### 2.5. Statistical Analysis

The data were analyzed using SPSS version 26.0 (IBM, New York, NY, USA). The Prodetect^TM^ buccal-nasal swabs RTK-Ag’s diagnostic performance was evaluated by comparing to the WHO-recognized Nasopharyngeal swabs RTK-Ag assay as the gold standard for diagnostic sensitivity and specificity determination. Person Chi-square (χ^2^) and Fisher Exact Tests were used to analyze the association between Nasopharyngeal swabs RTK-Ag assay and buccal-nasal swabs RTK-Ag from Prodetect^TM^. *p*-values less than 0.05 were considered to indicate a significant association.

## 3. Results

Study participants (*n* = 120) who had previously tested positive for SARS-CoV-2 by RTK-Ag test (nasopharyngeal) test performed at Emergency Department of Hospital Universiti Sains Malaysia (HUSM) were recruited with no age and gender limitation. Table 1 reports the baseline demographics of study participants who tested positive and negative for the buccal-nasal Prodetect^TM^ (Mediven Sdn Bhd, Penang, Malaysia) and nasopharyngeal RTK-antigen test such as Panbio™ COVID-19 Ag Rapid Test Device (Nasopharyngeal) (Abbott Rapid Diagnostics Jena GmbH, Jena, Germany) for SARS-CoV-2 (Table 1).

Out of 120 patients recruited, buccal–nasal Prodetect^TM^ result showed 108 true-positives, eight true negative and four false-negatives compared to nasopharyngeal RTK-Ag as a reference standard. The sensitivity and specificity of the paired RTK-Ag test in detecting the antigen by lateral flow were calculated at 96.4% and 100%, respectively. The test’s positive predictive value (PPV) is 100%, while the negative predictive value (NPV) is 66.7% (Table 2). Fisher exact tests showed the association between nasopharyngeal swabs RTK-Ag assay and buccal–nasal swabs RTK-Ag from Prodetect^TM^ is significant (*p*-values < 0.001) (Table 3).

## 4. Discussion

We report on our evaluation of SARS-CoV-2 antigen-detecting lateral flow device (LFD), focusing on the Prodetect^TM^ COVID-19 Antigen Rapid Self-Test, which has a sensitivity of 96.4% and specificity of 100%, using nasopharyngeal RTK-antigen test carried out by trained medical personnel for positive and negative status. The 100% positive predictive value (PPV) of Prodetect^TM^ means the probability of correctly identifying people who contracted the SARS-CoV-2 infections is total. Its negative predictive value (NPV), which means the probability of correctly identifying people who do not have a condition, is 66.7%.

The result showed that a self-conducted buccal and nasal RTK-antigen rapid test by the patients is comparable to the results obtained from a rapid test device conducted by trained medical personnel using a nasopharyngeal swab. The lower sensitivity for the kit may be accounted for by the timeline of the disease in patients recruited when the test was carried out. Most COVID-19 patients delayed their hospital visits after the first onset of symptoms due to fever or seasonal flu symptoms similarity. A prior study suggested Ag-RDTs showed higher sensitivity in patients within seven days after onset of symptoms than those in the later course of the disease [17]. This correlates with a study showing samples from patients within the first week after symptom onset contain the highest virus concentrations [18].

A systematic review and meta-analysis were carried out by Brümmer and colleagues [17] to analyze the accuracy of rapid antigen diagnostics for SARS-CoV-2 compared to nucleic acid detection from 133 analytical and clinical studies resulted in 214 clinical accuracy datasets with 112,323 samples. The meta-analysis showed that the pooled Ag-RDT sensitivity and specificity were 71.2% (95% CI 68.2% to 74.0%) and 98.9% (95% CI 98.6% to 99.1%), respectively. The pooled sensitivity for the 61 different types of Ag-RDT kit reported is comparably low to the golden standard of SARS-CoV-2 diagnosis, RT-qPCR with pooled diagnostic sensitivity and specificity using Reitsma’s bivariate models at 92.7% (95% CI 88.3 to 95.6%) and 92.9% (95% CI 87.2 to 96.2%), respectively [19].

The WHO has released interim guidance for Ag-RDTs to meet the minimum performance requirement of 80% sensitivity and 97% specificity to diagnose active SARS-CoV-2 infection. WHO also recommended that Ag-RDTs are best performed in symptomatic individuals with high viral load early in infection, especially in low-resource settings without rapid access to nucleic acid amplification technology (NAAT) [20]. The comparable data between Ag-RDT and nucleic acid detection by RT-qPCR is indicative of Ag-RDT ability to detect SAR-CoV-2 infection and its ability to control the pandemic in low-resource settings by the general public. The utilization of Ag-RDT in the community, despite its limited sensitivity, is likely to identify highly contagious individuals, substantially decreasing transmission rapidly. Apart from that, using Ag-RDTs as the first-line screening test, especially in emergency departments or COVID testing outpatient clinics, will provide a more rapid and cost-effective diagnosis in a high prevalence area than NAAT detection.

A reliable rapid antigen test such as Prodetect^TM^ and preventative measures such as continuous mask-wearing, social distancing, hand hygiene, and any other actions advised by the World Health Organization (WHO) can support a safer return post-pandemic daily life by swiftly identifying and isolating infectious individuals. In countries with high infectivity rates such as Malaysia, with its’ R-Naught (Ro) fluctuating between 0.8 to 1.0 from August to October 2021 [21], reliable, accessible, and affordable diagnostic testing for SARS-CoV-2 is a critical component to a comprehensive prevention and control strategy for COVID-19.

## 5. Conclusions

The sensitivity and specificity of the paired RTK-Ag test in detecting the antigen were at 96.4% and 100%, respectively. The test’s positive predictive value (PPV) is 100%, while the negative predictive value (NPV) is 66.7%. This showed that using a self-conducted buccal and nasal RTK-antigen rapid test is comparable to a rapid test device conducted by trained medical personnel using a nasopharyngeal swab. This indicates that the general public can rapidly utilize self-test Ag-RDT to control the pandemic in low-resource settings.

## Figures and Tables

**Table 1:** Baseline demographic of the patients and the results of nasopharyngeal swab and paired nasal and oral swabs.

No (%)					
Patients (*n* = 120)					
RTK Result	True Positives	False Negatives	False Positives	True Negatives	Total
RTK (paired buccal + nasal swab) *^1^					
Prodetect^TM^	108 (90)	4 (3.3)	0	8 (6.7)	120 (100)
RTK-Ag kit (nasopharyngeal swab) *^2^					
Abbott ^#^	100	0	0	8	108
Unknown	12	0	0	0	12
Total	112 (93.3)	0	0	8 (6.67)	120 (100)
**Characteristics**					
Age					
1–17	10 (8.3)	0	0	0	10 (8.3)
18–29	13 (10.8)	2 (1.7)	0	0	15 (12.5)
30–49	38 (31.7)	2 (1.7)	0	2 (1.7)	42 (35)
50 and above	44 (36.7)	0	0	5 (4.2)	49 (40.8)
Unknown	3 (2.5)	0	0	1 (0.8)	4 (3.3)
Total					120 (100)
Sex					
Female	59 (49.2)	2 (1.7)	0	4 (3.3)	65 (54.2)
Male	49 (40.8)	2 (1.7)	0	4 (3.3)	55 (45.8)
Total					120 (100)

*^1^—self-test by patients; *^2^—sample collected and ran by medical personnel; ^#^ Panbio™ COVID-19 Ag Rapid Test Device (Nasopharyngeal) (Abbott Rapid Diagnostics Jena GmbH, Jena, Germany).

**Table 2:** Buccal-nasal Prodetect^TM^ Nasopharyngeal RTK-Ag Crosstabulation.

			Nasopharyngeal RTK-Ag Test		Total
			Positive	Negative	
Prodetect^TM^	Positive	Count	108	0	108
		% within Prodetect^TM^	100%	0.0%	100.0%
		% within RTK	96.4%	0%	90.0%
		% of Total	90.0%	0.0%	90.0%
	Negative	Count	4	8	12
		% within Prodetect^TM^	33.3%	66.7%	100.0%
		% within RTK	3.6%	100.0%	10.0%
		% of Total	3.3%	6.7%	10.0%
Total		Count	112	8	120
		% within Prodetect^TM^	93.3%	6.7%	100.0%
		% within RTK	100.0%	100.0%	100.0%
		% of Total	93.3%	6.7%	100.0%

**Table 3:** Result for Chi-Square test.

	Value	df	Asymp. Sig. (2-Sided)	Exact Sig. (2-Sided)	Exact Sig. (1-Sided)
Pearson Chi-Square	77.143 ^a^	1	0.000		
Continuity Correction ^b^	66.801	1	0.000		
Likelihood Ratio	43.507	1	0.000		
Fisher’s Exact Test				0.000	0.000
Linear-by-Linear Association	76.500	1	0.000		
*n* of Valid Cases	120				

^a^ 1 cells (25.0%) have expected count less than 5. The minimum expected count is 0.80. ^b^ Computed only for a 2 × 2 table.

## Data Availability

Not applicable.
